# Programme Science in PEPFAR: a pathway to a sustainable HIV response

**DOI:** 10.1002/jia2.26244

**Published:** 2024-07-10

**Authors:** Michael J. A. Reid, Rebecca Bunnell, Emily Kainne Dokubo, John Nkengasong

**Affiliations:** ^1^ Bureau of Global Health Security and Diplomacy President's Emergency Plan for AIDS Relief Washington DC USA; ^2^ Institute of Global Health Sciences University of California at San Francisco San Francisco California USA; ^3^ U.S. Centers for Disease Control and Prevention Atlanta Georgia USA

1

Since its inception, the President's Emergency Plan For AIDS Relief (PEPFAR) has had an unprecedented impact on the global HIV epidemic. In the past 20 years, PEPFAR has saved more than 25 million lives, supporting over 20 million people with life‐saving treatment across 55 countries [1]. From the beginning, its programmes have relied on the best scientific insights and biomedical discoveries to advance the strategic HIV agenda. Moving forward, PEPFAR faces numerous important challenges in supporting partner countries to achieve the UNAIDS targets of ensuring that 95% of people with HIV know their status, 95% of those are on treatment and 95% of those are virologically suppressed [2], and achieving its goal of ending the HIV pandemic as a public health threat by 2030. As such, it is important to consider how Programme Science—*an approach that emphasizes the need for context‐specific evidence and knowledge generated on an ongoing basis, to inform the timely and strategic programmatic decisions* [3, 4]—can help ensure that PEPFAR programmes implement the right set of context‐specific interventions to achieve and sustain the changes essential for HIV prevention and treatment outcomes. In this paper, we detail (1) the evolving challenges and opportunities PEPFAR faces in achieving 95‐95‐95, (2) how *Programme Science* can be strategically applied to overcome these challenges, and (3) the necessary adaptations needed to sustain HIV programmes, informed by the best, contextually relevant science.

As PEPFAR and its partners intensify efforts to achieve the UNAIDS targets and sustain the progress achieved, they face a range of complex and urgent health challenges. Addressing these challenges requires a Programme Science approach that is responsive to diverse real‐world contexts—be they geographical, organizational or sociological [5].

One of the most urgent challenges PEPFAR programmes face is reaching those populations that are not effectively covered by existing services. This is especially true in many countries with high HIV burden, where there is a pressing and urgent need to devise innovative approaches to engage adolescents and young women who are at inordinate risk of HIV acquisition. Equally important is the provision of targeted interventions for specific key and priority groups like men who have sex with men and female sex workers, who often face significant barriers in accessing health services due to stigma, discrimination, and legal and human rights issues.

Another critical area of focus is supporting continuity of care for people living with HIV who have been linked to HIV services. Across PEPFAR programmes, over 450,000 persons experienced interruptions in treatment at some point in the third quarter of 2023 [6]. As such, supporting clients to stay engaged in lifelong care necessitates the identification and implementation of a tailored mix of biomedical, behavioural and structural interventions to ensure the continued provision and uptake of HIV treatment services. These interventions must be adaptable to suit the diverse needs of different populations in various locations.

Additionally, HIV programme must address the system‐level challenges necessary to ensure that existing HIV services have an enduring impact and are optimally integrated into government‐run programmes. As such, programmes must determine how to strengthen health systems so that they meet the individualized needs of clients with co‐morbid diseases like hypertension [7], while also working with partner governments to determine opportunities for synergy and integration of HIV services into primary care systems.

Advancing PEPFAR's commitment to health equity while determining how and where to deploy new biomedical tools also raises a unique set of Programme Science questions [8, 9]. Decisions about the deployment of tools like long‐acting HIV prevention and treatment must include consideration of context‐specific factors, including end‐user preferences [10], service delivery infrastructure [11] and resource availability.

Faced with these different and diverse challenges and opportunities, the new PEPFAR five‐year strategic plan calls for an integration of science directly into programme planning and execution. Moreover, it will require a dynamic approach to how scientific insights are derived directly from programme experiences rather than only translating research into policy [12]. In the past, PEPFAR's policy decisions have often been guided by applying proven strategies from one context to another and leveraging existing hypothesis‐driven science to inform planning, applying insights from science to policy and programmes. Notable examples include the introduction of the “Treatment as Prevention” strategy [13] and scaling pre‐exposure prophylaxis interventions [14]. Policies for both were developed based on a substantial body of evidence that pre‐existed policy formulation. However, this approach relied on scientific evidence being readily available to answer PEPFAR's strategic questions [15].

To realize the goals outlined in PEPFAR's strategic plan requires a shift in how programmes inform science and how and when science is used to shape its programmes. It demands that HIV programmes play a primary role in identifying how science is leveraged to close gaps in HIV service provision and to determine how best to scale new integrated service delivery models or potentially transformative biomedical tools. The focus needs to be on generating insights relevant to specific contexts that are also generalizable to different settings while prioritizing health equity.

Moving forward, PEPFAR is planning future initiatives across supported countries and regions to contribute to Programme Science, while also encouraging others working on the HIV response to expand their contributions to Programme Science too. PEPFAR's upcoming Country Operational Planning Guidance will highlight the importance of Programme Science for PEPFAR‐supported country and regional programmes to prioritize in their operational planning process, aimed at addressing strategic priorities and programmatic gaps specific to each country or region. Incorporating Programme Science into operational planning, PEPFAR can better address identified gaps in knowledge and practice, ensuring that interventions are both scientifically grounded, pragmatically viable for diverse global contexts and responsive to local strategic priorities.

A critical next step involves empowering HIV programmes in each country in partnership with Ministries of Health, National Public Health Institutions, local academic partners and community leadership to identify the gaps where Programme Science can help ensure that evidence informs programmes on the timeline and at the scale necessary to drive impact. Creating the capacity to use Programme Science to inform strategic planning and resource allocation can ensure that programmes are continually responsive to the evolution of the HIV pandemic and that the needed biomedical and clinical tools, as well as essential resources, are available. Zambia offers a useful example of how this can work; a data hub run by the Ministry of Health with technical and limited financial support from PEPFAR is using rigorous analytic approaches to interrogate programme data to understand how and where programme improvements are likely to be most impactful. The Zambia example also illustrates how embedding Programme Science into how PEPFAR country programmes operate can also help advance PEPFAR's commitment to sustainable development, strengthened public health systems and transformative partnerships [16]. This approach not only addresses current challenges but also lays the groundwork for long‐term success, ensuring that programmes continue to prioritize equity and impact.

As PEPFAR enters a new phase in its response to the HIV pandemic, it is important to ensure that policies and strategies are both evidence‐based and tailored to the unique challenges of different communities and regions. As such, PEPFAR is committed to taking necessary steps to ensure that HIV programmes are empowered and have the capacity to undertake the kinds of analyses necessary for Programme Science to lead to programme improvement. We are explicitly designing new processes and mechanisms into our funding and policy guidance that foster the enabling environment for science and innovation necessary to accelerate progress. These changes can ensure that each country realizes the goal of 95‐95‐95 and that PEPFAR programmes continue to adapt to the evolving pandemic while also empowering the relevant local stakeholders to take the lead in using science to improve programmes.

## COMPETING INTERESTS

The authors declare no competing interests.

## AUTHORS’ CONTRIBUTIONS

RB and MJAR conceived of the presented idea. The first draft was written by MJAR, and all other authors revised and edited subsequent drafts. All authors reviewed the final, submitted draft.

## References

[1] PEPFAR latest global results. Washington, DC: US Government; 2023.

[2] UNAIDS . The path that ends AIDS. Geneva; 2023.

[3] Aral SO , Blanchard JF . The Programme Science initiative: improving the planning, implementation and evaluation of HIV/STI prevention programmes. Sex Transm Infect. 2012; 88(3):157–159. 22363021 10.1136/sextrans-2011-050389

[4] Becker M , Mishra S , Aral S , Bhattacharjee P , Lorway R , Green K , et al. The contributions and future direction of Programme Science in HIV/STI prevention. Emerg Themes Epidemiol. 2018; 15:7. 29872450 10.1186/s12982-018-0076-8 PMC5972407

[5] Nachega JB , Musoke P , Kilmarx PH , Gandhi M , Grinsztejn B , Pozniak A , et al. Global HIV control: is the glass half empty or half full? Lancet HIV. 2023; 10(9):e617–e622. 37506723 10.1016/S2352-3018(23)00150-9 PMC10733629

[6] PEPFAR Panorama Spotlight. Washington, DC: PEPFAR; 2023.

[7] Nkengasong J , Ruffner M , Bartee M , Katz IT , Reid MJA . Sustaining the HIV/AIDS response: PEPFAR's vision. J Int AIDS Soc. 2023; 26(12):e26192. 38031907 10.1002/jia2.26192 PMC10687756

[8] Delany‐Moretlwe S , Hughes JP , Bock P , Ouma SG , Hunidzarira P , Kalonji D , et al. Cabotegravir for the prevention of HIV‐1 in women: results from HPTN 084, a phase 3, randomised clinical trial. Lancet. 2022; 399(10337):1779–1789. 35378077 10.1016/S0140-6736(22)00538-4 PMC9077443

[9] Landovitz RJ , Donnell D , Clement ME , Hanscom B , Cottle L , Coelho L , et al. Cabotegravir for HIV prevention in cisgender men and transgender women. N Engl J Med. 2021; 385(7):595–608. 34379922 10.1056/NEJMoa2101016 PMC8448593

[10] Kerkhoff AD , Muiruri C , Geng EH , Hickey MD . A world of choices: preference elicitation methods for improving the delivery and uptake of HIV prevention and treatment. Curr Opin HIV AIDS. 2023; 18(1):32–45. 36409315 10.1097/COH.0000000000000776 PMC9772083

[11] Geng EH , Nash D , Phanuphak N , Green K , Solomon S , Grimsrud A , et al. The question of the question: impactful implementation science to address the HIV epidemic. J Int AIDS Soc. 2022; 25(4):e25898. 35384312 10.1002/jia2.25898 PMC8982316

[12] McClarty LM , Becker ML , Garcia PJ , Garnett GP , Dallabetta GA , Ward H , et al. Programme Science: a route to effective coverage and population‐level impact for HIV and sexually transmitted infection prevention. Lancet HIV. 2023; 10(12):e825–e834. 37944547 10.1016/S2352-3018(23)00224-2

[13] Russell A , Verani AR , Pals S , Reagon VM , Alexander LN , Galloway ET , et al. Impact of HIV treat‐all and complementary policies on ART linkage in 13 PEPFAR‐supported African countries. BMC Health Serv Res. 2023; 23(1):1151. 37880619 10.1186/s12913-023-09702-2 PMC10598983

[14] Eakle R , Gomez GB , Naicker N , Bothma R , Mbogua J , Cabrera Escobar MA , et al. HIV pre‐exposure prophylaxis and early antiretroviral treatment among female sex workers in South Africa: results from a prospective observational demonstration project. PLoS Med. 2017; 14(11):e1002444. 29161256 10.1371/journal.pmed.1002444 PMC5697804

[15] Parkhurst J , Weller I , Kemp J . Getting research into policy, or out of practice, in HIV? Lancet. 2010; 375(9724):1414–1415. 20417842 10.1016/S0140-6736(10)60585-5

[16] State Do. PEPFAR's five‐year strategy: fulfilling America's promise to end the HIV/AIDS pandemic by 2030. 2022.

